# Supplementary material for: STAT3 and HIF1α cooperatively mediate the transcriptional and physiological responses to hypoxia
Source: Cell Death Discov. 2023 Jul 5;9:226. doi: 10.1038/s41420-023-01507-w (PMC10323006; doi:10.1038/s41420-023-01507-w)
Supplement: Supplementary file 1 — Supplementary figures [file 41420_2023_1507_MOESM1_ESM.pdf]

Supplementary Figures

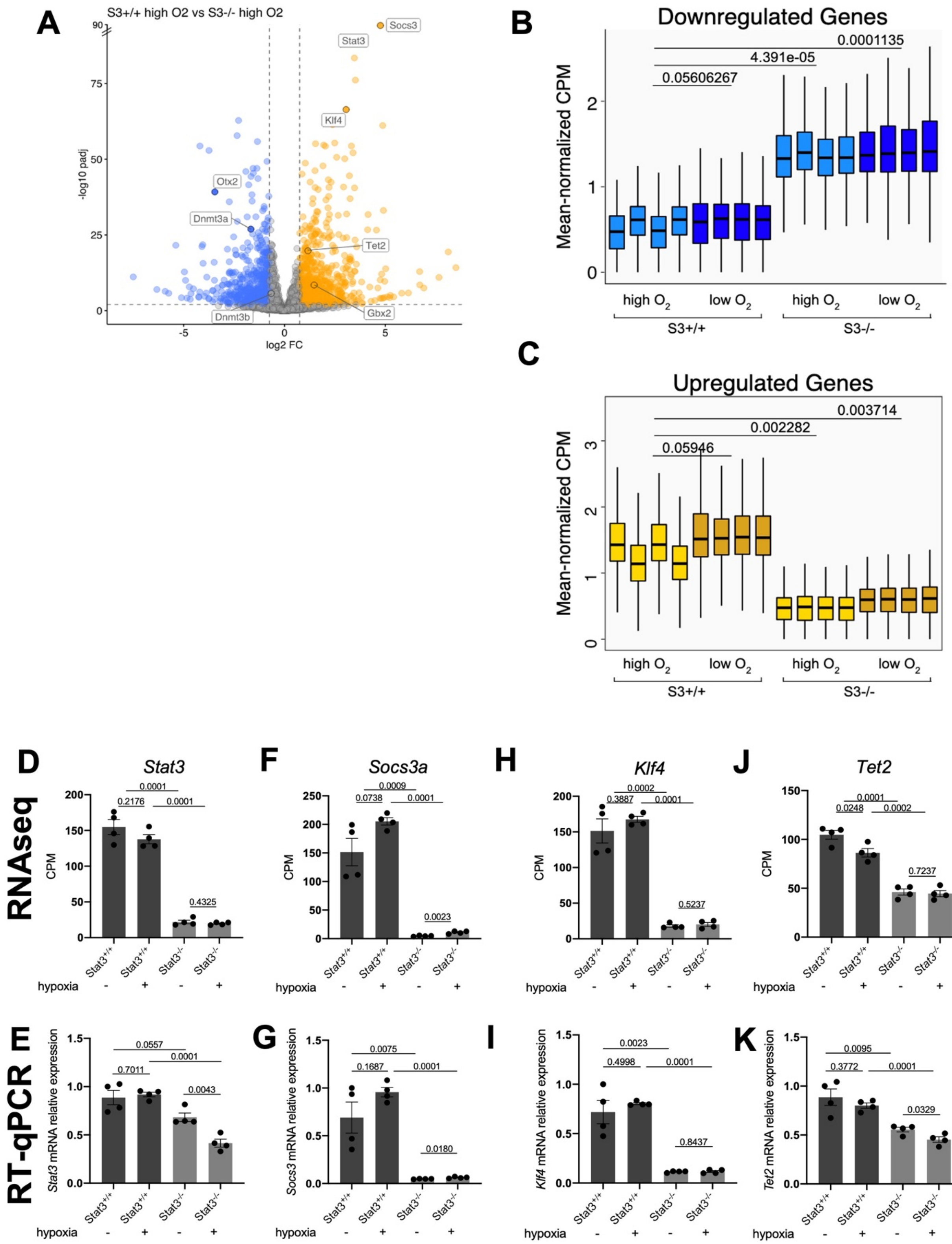

Fig. S1: **Transcriptome analysis by RNAseq**. A: Genes that were differentially expressed (log2[fold change (FC)] > + 0.75 or < −0.75; q-value < 0.01, Benjamini–Hochberg adjustment, as indicated by dashed lines) between *Stat3*$^{+/+}$ and Stat3$^{-/-}$ cells; n = 4 biological replicates. B: Expression levels of genes that were downregulated in *Stat3*$^{+/+}$ relative to *Stat3*$^{-/-}$ cells. C: Expression levels of genes that were upregulated in *Stat3*$^{+/+}$ relative to *Stat3*$^{-/-}$ cells. Each boxplot shows the 1st, 2nd and 3rd quartiles; the whiskers show the minimum and maximum values. D,E: *Stat3* expression in *Stat3*$^{+/+}$ and *Stat3*$^{-/-}$ cells incubated either in normoxia and hypoxia measured with RNAseq (D) and RT-qPCR (E). F,G: *Socs3* expression in *Stat3*$^{+/+}$ and *Stat3*$^{-/-}$ cells incubated either in normoxia and hypoxia measured with RNAseq (F) and RT-qPCR (G). H,I: *Klf4* expression in *Stat3*$^{+/+}$ and *Stat3*$^{-/-}$ cells incubated either in normoxia and hypoxia measured with RNAseq (H) and RT-qPCR (I). J,K: *Tet2* expression in *Stat3*$^{+/+}$ and *Stat3*$^{-/-}$ cells incubated either in normoxia and hypoxia measured with RNAseq (J) and RT-qPCR (K). Mean±SEM.

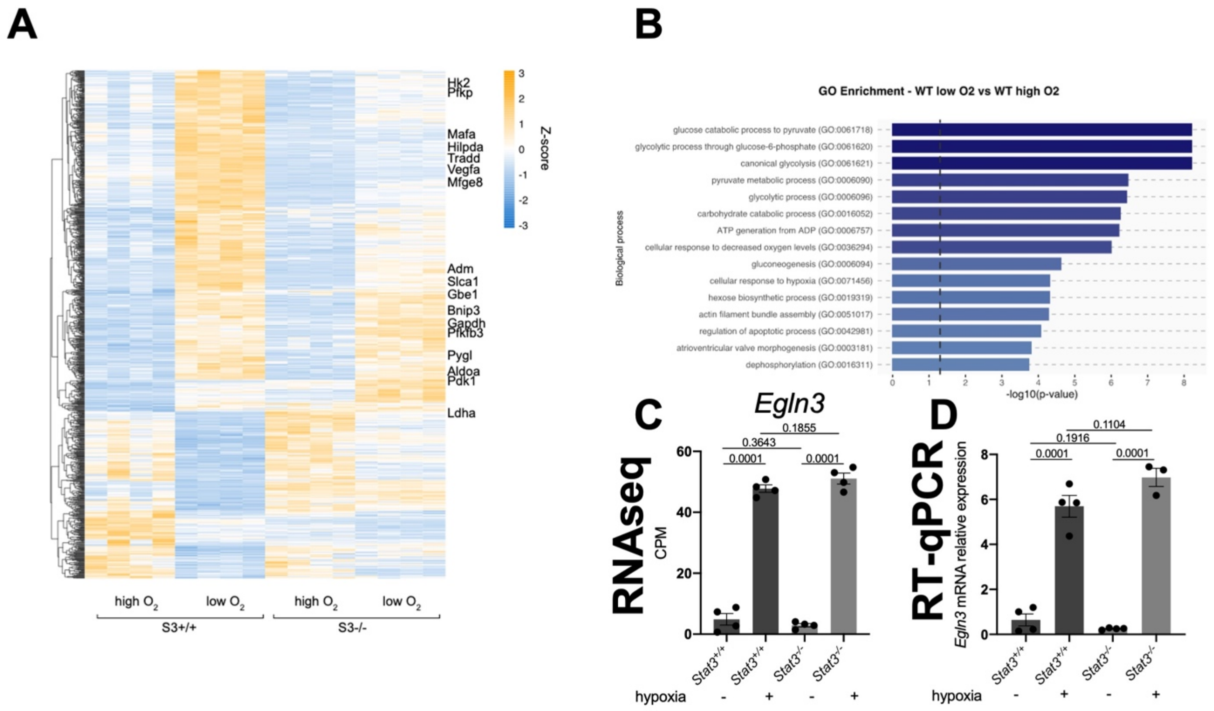

Fig. S2: **hypoxia and CoCl$_2$ induce HIF1α target genes in *Stat3*$^{+/+}$ cells.** A: heatmap of RNAseq data reporting the expression of genes that are affected by hypoxia in *Stat3*$^{+/+}$ cells compared to *Stat3*$^{+/+}$ normoxic cells. B: GO enrichment between normoxic and hypoxic *Stat3*$^{+/+}$ cells. C,D: expression level of *Egln3* in normoxic and hypoxic *Stat3*$^{+/+}$ and *Stat3*$^{-/-}$ mESCs obtained from RNAseq data (C) and from RT-qPCR (D). Mean±SEM.

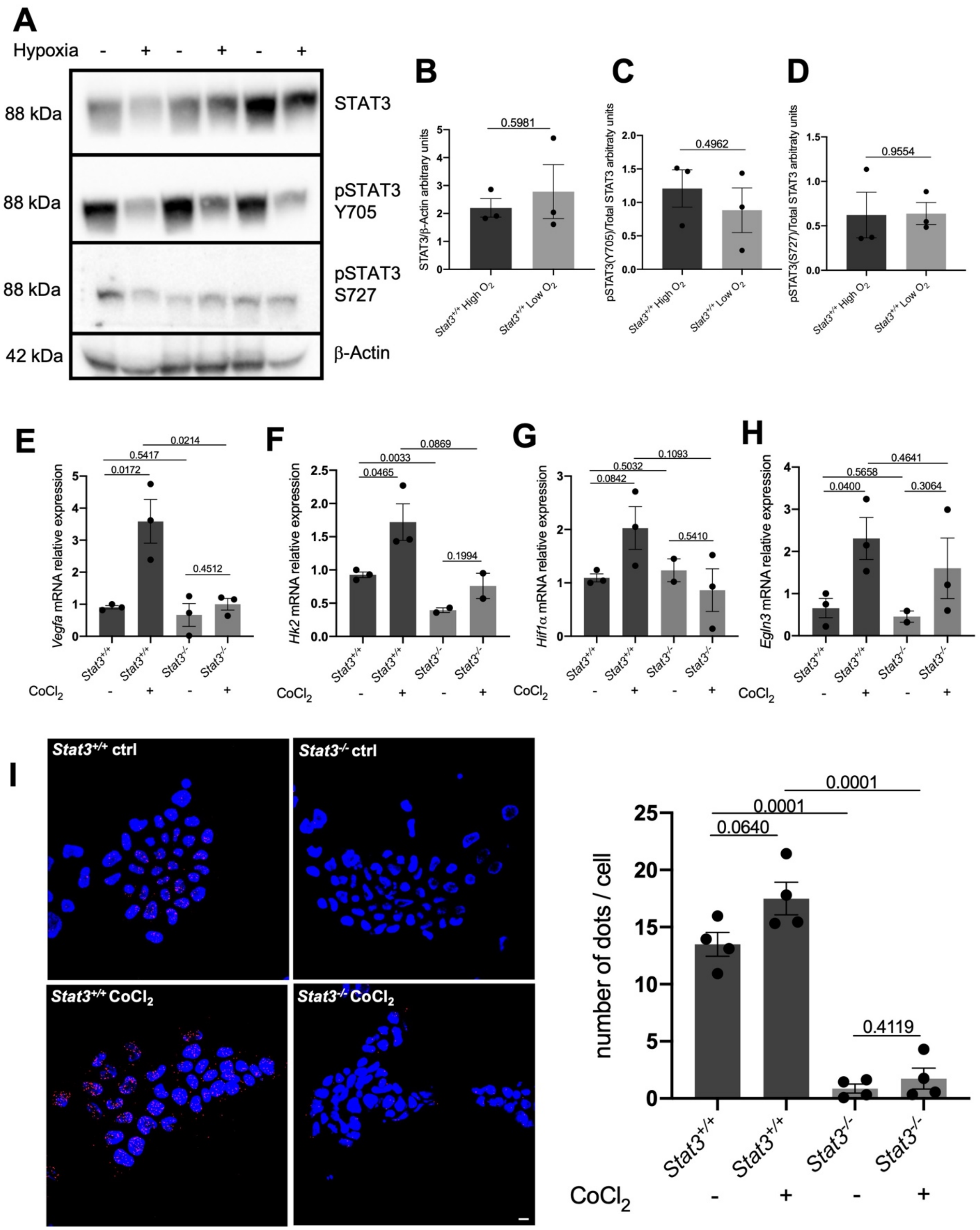

Fig. S3: **Hypoxia does not affect STAT3 expression nor its phosphorylations.** A-D: western blot pictures and quantification of total STAT3 (B), pSTAT3 Y705 (C) and pSTAT3 S727 (D) in murine *Stat3*$^{+/+}$ ESCs kept in high or low oxygen

tensions for 24 hours. E: RT-qPCR analysis of *Vegfa* mRNA expression in *Stat3*$^{+/+}$ and *Stat3*$^{-/-}$ ESCs incubated either with DMSO or $CoCl_2$. F: RT-qPCR analysis of *Hk2* mRNA expression in *Stat3*$^{+/+}$ and *Stat3*$^{-/-}$ ESCs incubated either with DMSO or $CoCl_2$. G: RT-qPCR analysis of *Hif1α* mRNA expression in *Stat3*$^{+/+}$ and *Stat3*$^{-/-}$ ESCs incubated either with DMSO or $CoCl_2$. H: RT-qPCR analysis of *Egln3* mRNA expression in *Stat3*$^{+/+}$ and *Stat3*$^{-/-}$ ESCs incubated either with DMSO or $CoCl_2$. I: PLA of STAT3 and HIF1α in *Stat3*$^{+/+}$ and *Stat3*$^{-/-}$ ESCs incubated either with DMSO or $CoCl_2$. Representative pictures and quantification. Scale bar = 200 µm. Mean±SEM.

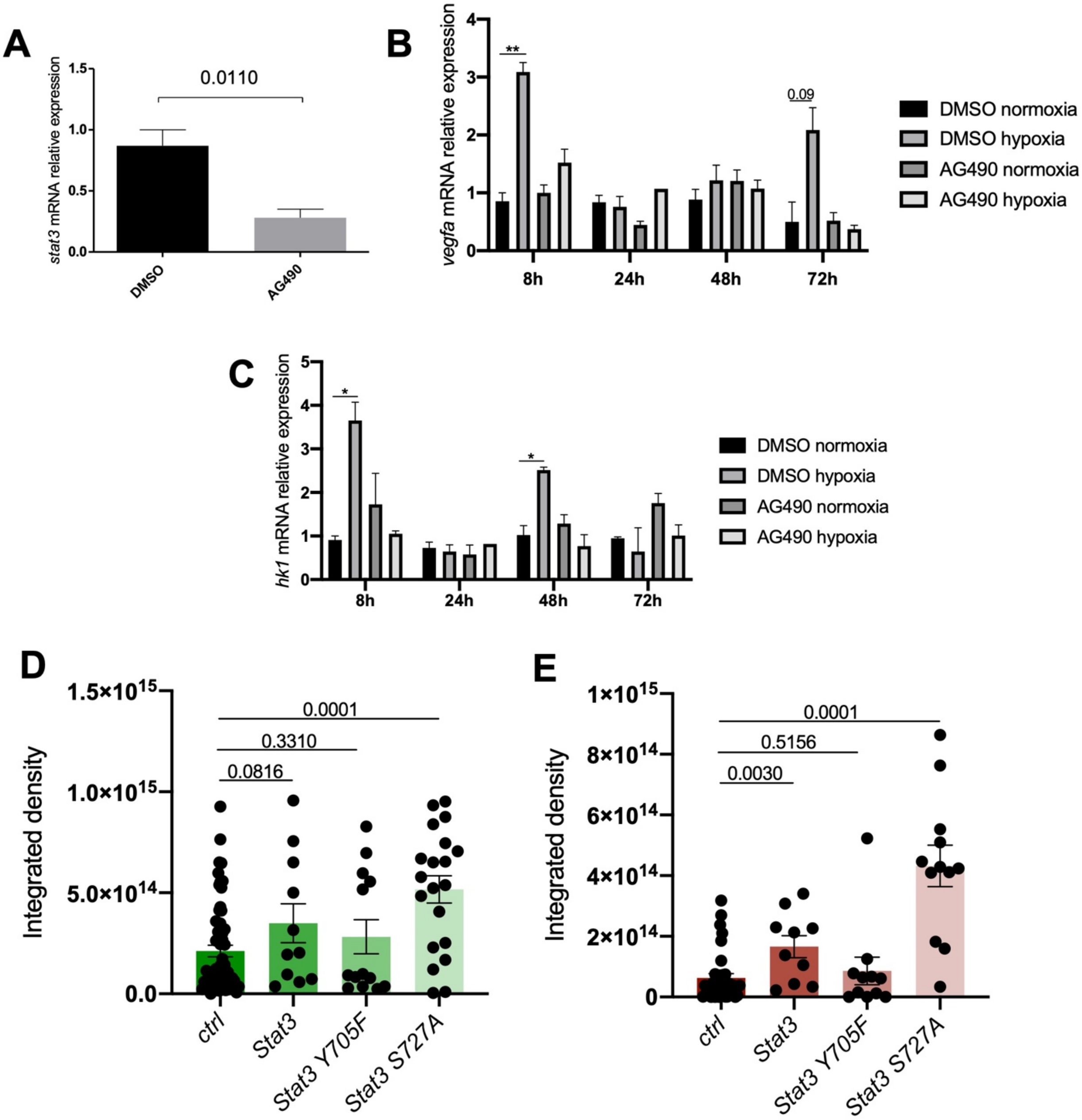

Fig. S4: **AG490 blocks transcriptional response to hypoxia.** A: *stat3* expression level of 6-dpf larvae treated with either DMSO and 50 µM AG490 from 3 dpf to 6 dpf. n = 3 biological replicates. B: *vegfa* expression of larvae incubated in normoxia or hypoxia and treated with either DMSO and 50 µM AG490 for 8, 24, 48 and 72 hours. n = 3 biological replicates. C: *hk1* expression of larvae incubated in normoxia or hypoxia and treated with either DMSO and 50 µM AG490 for 8, 24, 48 and 72 hours. n = 3 biological replicates. D: EGFP fluorescence quantification of *Tg(7xStat3-Hsv.Ul23:EGFP)^{ia28}* reporter larvae injected with control solution (n = 59), *Stat3* (n = 11), *Stat3 Y705F* (n = 13), *Stat3 S727A* (n = 20) mRNAs. E: mCherry fluorescence quantification of *HRE:mCHerry* reporter larvae injected with control solution, *Stat3, Stat3 Y705F, Stat3 S727A* mRNAs. Mean±SEM.

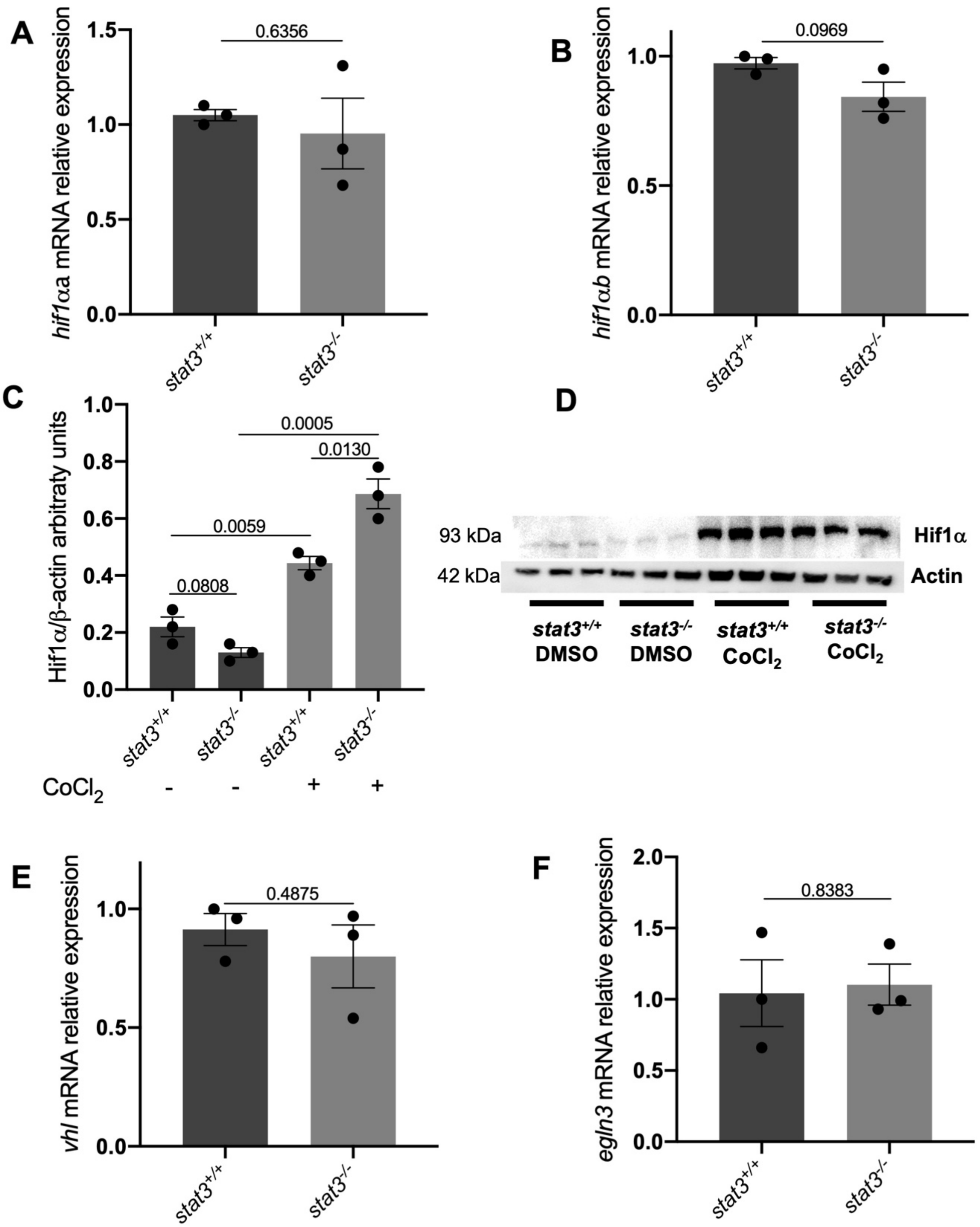

Fig. S5: ***hif* genes and Hif1α stabilization are not affected in *stat3* knock-out larvae.** A: *hif1αa* expression in 6-dpf *stat3*$^{+/+}$ larvae compared to *stat3*$^{-/-}$ larvae. B: *hif1αb* expression in 6-dpf *stat3*$^{+/+}$ larvae compared to *stat3*$^{-/-}$ larvae. n = 3 biological replicates. C,D: western blot analysis of Hif1α protein in *stat3*$^{+/+}$ and *stat3*$^{-/-}$ 6-dpf larvae treated either with DMSO or $CoCl_2$. Quantification (C) and representative pictures (D). n = 3 biological replicates. E: *vhl* expression in 6-dpf *stat3*$^{+/+}$

larvae compared to *stat3*$^{-/-}$ larvae. n = 3 biological replicates. F: *phd3* expression in 6-dpf *stat3*$^{+/+}$ larvae compared to *stat3*$^{-/-}$ larvae. n = 3 biological replicates. Mean±SEM. **p<0.01, ***p<0.001, ns=not significant.

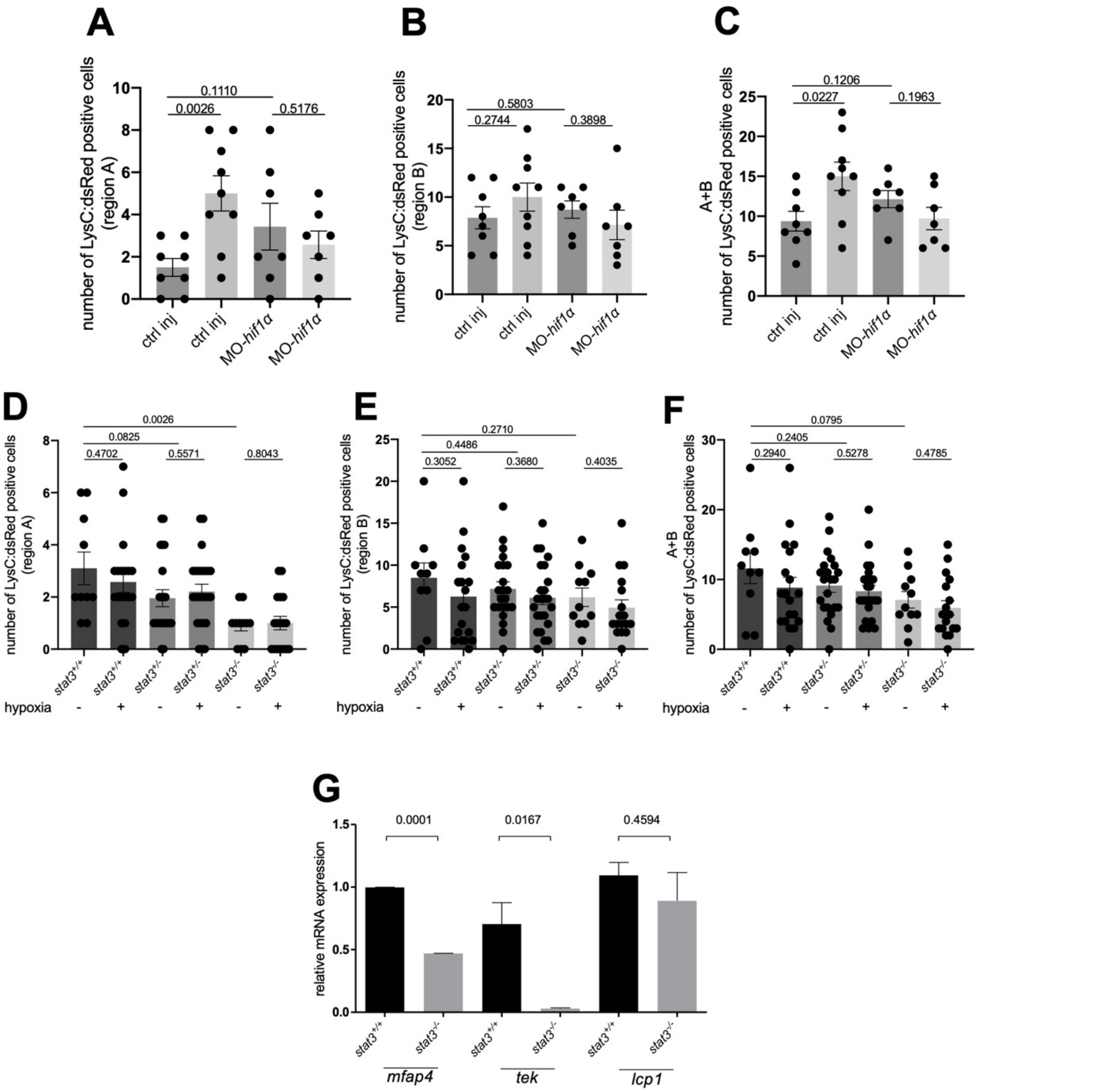

Fig. S6: **Analysis of macrophage mobilization in *stat3* mutants.** A-C: count of macrophages in region A (A), in region B (B) and in total (C) in ctrl and MO-*hif1a* injected larvae after incubation either in normoxia and in hypoxia. n = 8 ctrl inj normoxia; 9 ctrl inj hypoxia; 7 MO-*hif1α* normoxia; 8 MO-*hif1α* hypoxia (larvae used for this experiment came from three independent breeding between wild type zebrafish) D-F: count of macrophages in region A (D), in region B (E) and in total (F) in *stat3*$^{+/+}$, *stat3*$^{+/-}$ and *stat3*$^{-/-}$ larvae incubated either in normoxia and in hypoxia. n = 11 *stat3*$^{+/+}$ normoxia; 16 *stat3*$^{+/+}$ hypoxia; 22 *stat3*$^{+/-}$ normoxia; 23 *stat3*$^{+/-}$ hypoxia; 11 *stat3*$^{-/-}$ normoxia; 17 *stat3*$^{-/-}$ hypoxia (larvae used for this experiment

came from three independent breeding between *stat3*$^{+/-}$ zebrafish). G: *mfap4, tek* and *lcp1* expression in *stat3*$^{+/+}$ and *stat3*$^{-/-}$ 6-dpf larvae. n = 3 biological replicates. Mean±SEM.

**Gene**	**Forward sequence**	**Reverse sequence**	**Reference**
zgapdh	GTGGAGTCTACTGGTGTCTTC	GTGCAGGAGGCATTGCTTACA	Peron *et al*. (2020)
zstat3	TGCCACCAACATCCTAGTGT	GCTTGTTTGCACTTTTGACTGA	Peron *et al*. (2020)
zsocs3a	GGAAGACAAGAGCCGAGACT	GCGATACACACCAAACCCTG	Peron *et al*. (2020)
zhif1aa	AGATCCTGTTCTCACGCTGG	GTTGTAGATGGCCTGTGGAG	This paper
zhif1ab	GCGACGCCATTATCTCTCTG	AGTTTGGTCAGAGCTGGGAT	This paper
zepas1a	CCTACGACATGGGCGAAATA	GTCGCCTCTTCAAACTCTGC	This paper
zepas1b	GCCTATGGAGAACTTTGTGCA	TGCCGAAGTTTCTTCCACAC	This paper
zhif1al	AGAGCACACTTACCCACACA	ATGGGTGAGGTATGGGTTCG	This paper
zhif1al2	TGGATCTGATCGGAAGGAGC	CCAACCAGTCAGTTTCGTGT	This paper
zarnt	GGATTTCGCACACCCAGAAG	GGGTTCTGGAAGGTGAAGGA	This paper
zarnt2	GCAGTGCACCTTTGGATCAC	TGACCTGCTTCTGTGTCTTCA	This paper
zvegfa	AAAAGAGTGCGTGCAAGACC	TTTCGTGTCTCTGTCGGGAC	This paper
zhk1	TCACACCAGAGCTGCTTACA	AGCGAAAAGAGACAATGGCA	This paper
zphd3	CGGAGGAACATCAGAGGAGA	CCACCATTGCCTTTGACCTC	This paper
zmfap	TGCTCTCAGATGGGAAAGATG	GCCAGTATTCTCCCTCCACA	Gerri *et al.* (2017)
ztek	AGCTCCAGGAACACTGAGGA	ATGTGGAGCTGCTGTGTCTG	Gerri *et al.* (2017)
zlcp1	CGGAAGGCCATCAATAAGAA	CCTTCTCCAGAGCCTTGTTG	Gerri *et al.* (2017)
zflna	ATGGTGGTGATGAGATCCCG	TACAGGTCACCTTGCCCTTC	This paper
mActb	CTAAGGCCAACCGTGAAAAG	ACCAGAGGGCATACAGGGACA	Carbognin *et al.* (2016)
mStat3	TGTTGGAGCAGCATCTTCAG	GAGGTTCTCCACCACCTTCA	Carbognin *et al.* (2016)
mSocs3	ATTTCGCTTCGGGACTAGC	AACTTGCTGTGGGTGACCAT	Carbognin *et al.* (2016)
mVegfa	CTGTAACGATGAAGCCCTGGAG	TGGTGAGGTTTGATCCGCAT	Longchamp *et al.* (2018)

mHk1	TGAAGGGCGCATTACTCCAG	CAGGCGGGTCAAGATTTCCT	This Paper
mHk2	GAGGGTGGAGATGGAGAACC	CAGGGGAACGAGAAGGTGAA	This Paper
mPfkp	CTGAAGGAGCAATCGACATGC	CAAATGCAGATGGGGTCCCT	This Paper
mHilpda	CTGGGCATCATGTTGACCCT	TGTGTGTTGGCTAGCTGACC	This Paper
mPhd3	CTTCCTCCTGTCCCTCATCG	ATACAGCGGCCATCACCATT	Betto *et al*. (2021)
mHif1α	GAAGTGCACCCTAACAAGCC	GGGTTCACAAATCAGCACCA	This Paper

Table 1: list of primers used for RT-qPCR (5' → 3' sequences).
