# Supplementary figures and images for: STAT3 and HIF1α cooperatively mediate the transcriptional and physiological responses to hypoxia
Source: Cell Death Discov. 2023 Jul 5;9:226. doi: 10.1038/s41420-023-01507-w (PMC10323006; doi:10.1038/s41420-023-01507-w)

Figure 2

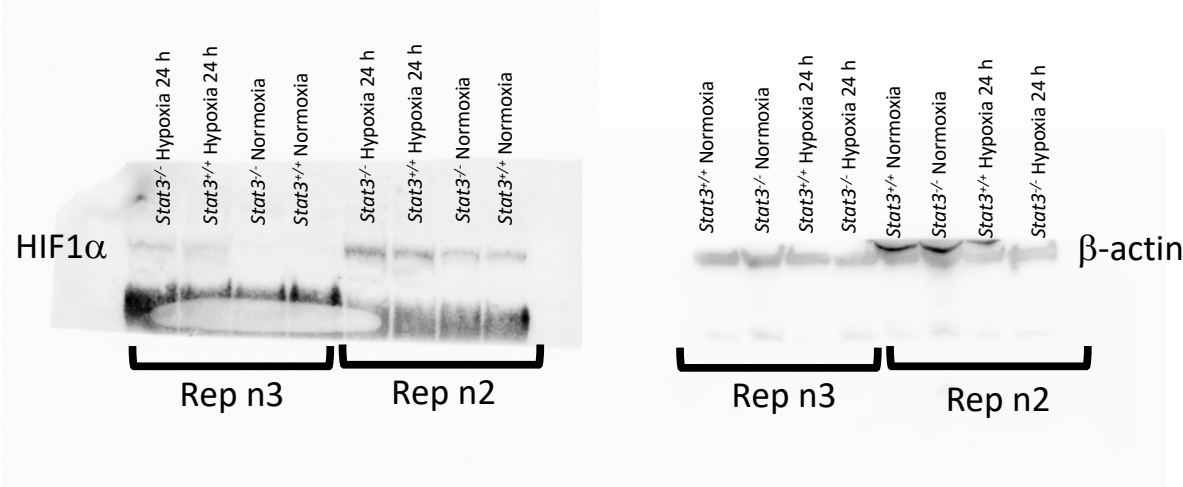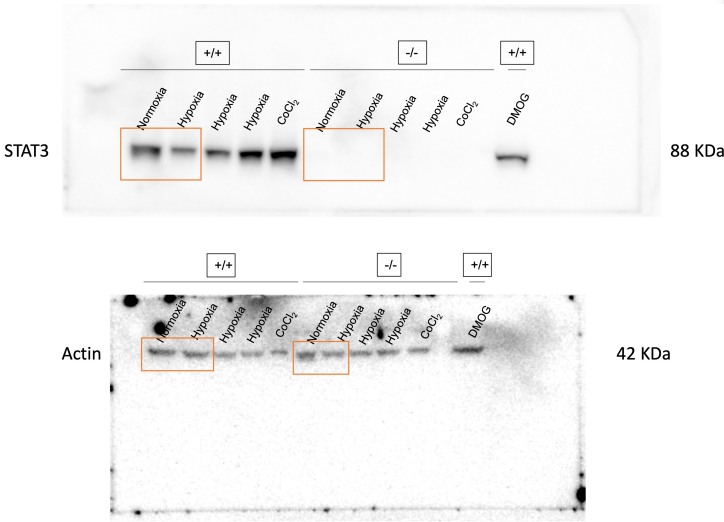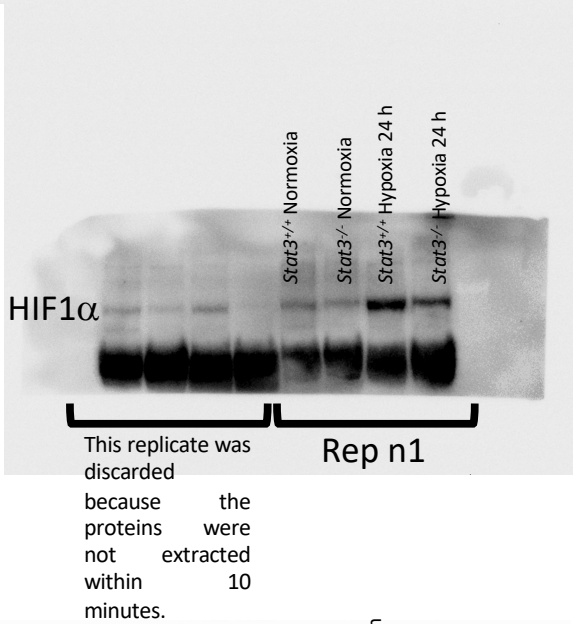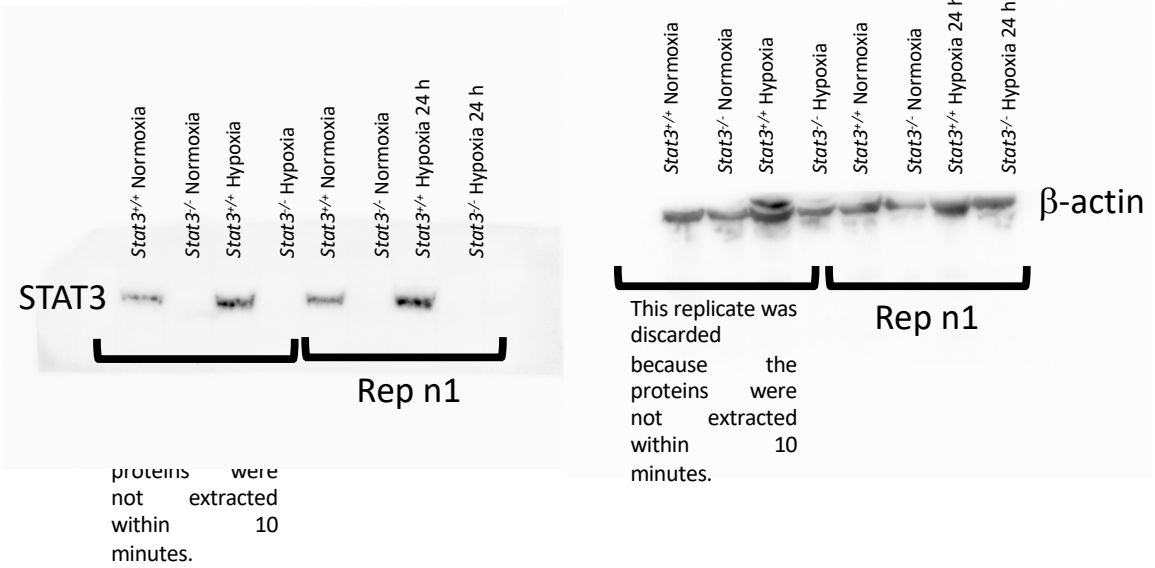

Supplementary Figure 2D

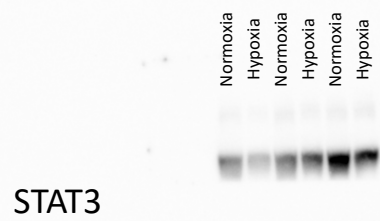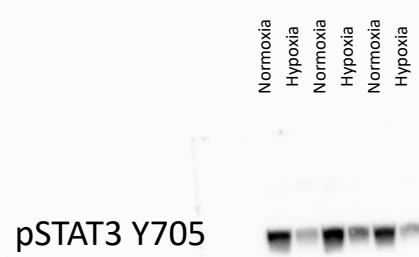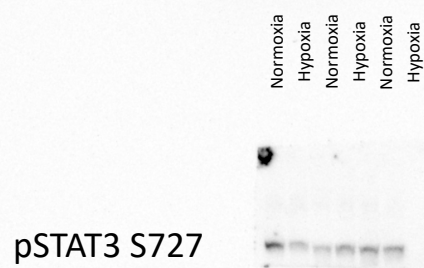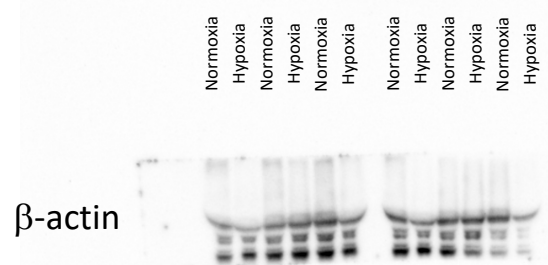

Supplementary Figure 5C

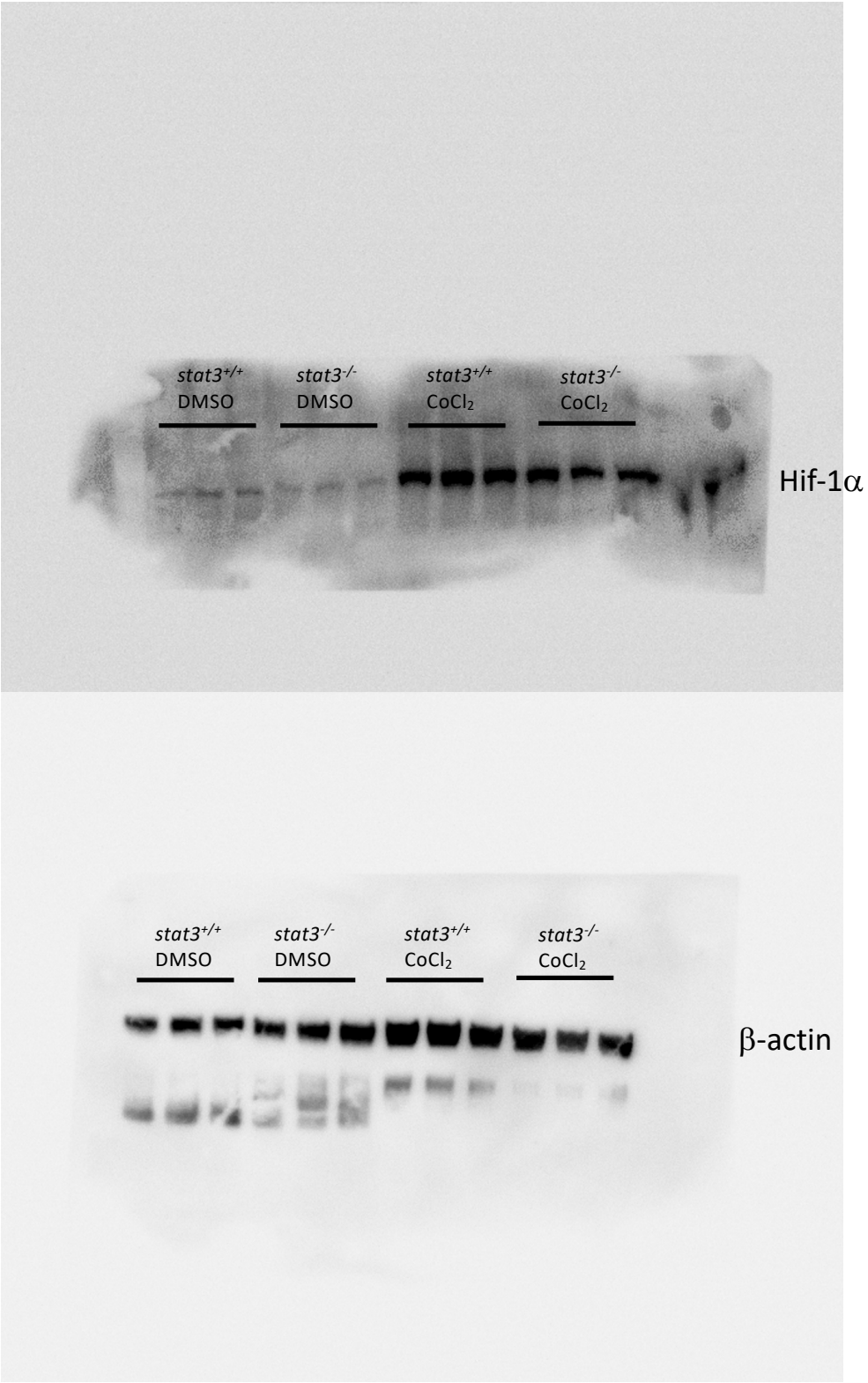

Supplement: Supplementary file 2 — Original Data File [file 41420_2023_1507_MOESM2_ESM.pdf]
